# Incorporating environmental variables as precursor background variables of the theory of planned behavior to predict quitting-related intentions: a comparative study between adult and young adult smokers

**DOI:** 10.1186/s13690-018-0311-3

**Published:** 2018-11-01

**Authors:** Chung Gun Lee, Susan E Middlestadt, Dong-Chul Seo, Hsien-Chang Lin, Jonathan T Macy, Seiyeong Park

**Affiliations:** 1 Department of Physical Education, College of Education, Seoul National University, 1 Gwanak-ro, Gwanak-gu, Seoul, 08826 South Korea; 2 Department of Applied Health Science, School of Public Health-Bloomington, Indiana University, 1025 E. Seventh Street, Bloomington, Indiana 47405-7109 USA

**Keywords:** Theory of planned behavior, Cigarette smoking, Quitting intention, Subjective norm, Adults

## Abstract

**Background:**

As previous studies suggest that the theory of planned behavior (TPB) is open to the inclusion of further predictors, identifying a number of additional background variables within the context of the TPB may help improve the predictive power of the theory. The purpose of this study is to incorporate environmental variables as precursor background variables of the TPB to predict quitting-related intentions.

**Methods:**

This study consists of two sub-studies. Sub-study 1 and 2 analyzed different data sets and were conducted using the similar methodology for the comparison. A total of 395 Texas adult smokers (sub-study 1) and 379 university student smokers (sub-study 2) were analyzed using multiple structural equation modeling.

**Results:**

The extent of agreement with regulating smoking in public places had positive indirect effects on intention to quit through subjective norm among both Texas adult smokers (*β* = 0.03, *p* < .01) and university students (*β* = 0.01, *p* < .05), and through attitude among Texas adult smokers only (*β* = 0.02, *p* < .01). The number of smokers among 5 closest friends had negative indirect effect on intention to take measures to quit through subjective norm among Texas adult smokers (*β* = − 0.02, *p* < .05).

**Conclusions:**

The results of this study indicate that environmental variables need to be considered as precursor background variables of the TPB to predict quitting-related intentions.

## Background

Although the smoking prevalence in the USA has significantly decreased during the past three decades, the decline in smoking rate has halted during the past 5 years [1]. Moreover, approximately 20% of US adults were still smoking in 2010 [2]. This is a significant public health issue because cigarette smoking is a well-known risk factor of chronic diseases, such as cardiovascular diseases, pulmonary diseases, and lung cancer [3]. More effective and efficient smoking cessation interventions, therefore, should be developed based on appropriate theoretical frameworks because the design of interventions that generate desirable outcomes can best be achieved by thoroughly understanding theories [4]. In order to do this, more basic research is needed to test and develop particular theoretical frameworks [5].

The theory of planned behavior (TPB) is one of the most frequently used theoretical frameworks for explaining behavioral intentions [6], including intention to quit smoking [7–14]. The TPB focuses on theoretical constructs reflecting individual’s motivational and cognitive factors as strong predictors of performance of the behavior. The TPB assumes the most proximal determinant of the behavior is intention to perform a behavior, which, in turn, is strongly affected by attitude and subjective norm toward a behavior and perceived behavioral control over performance of a behavior [15].

**Fig. 1:**
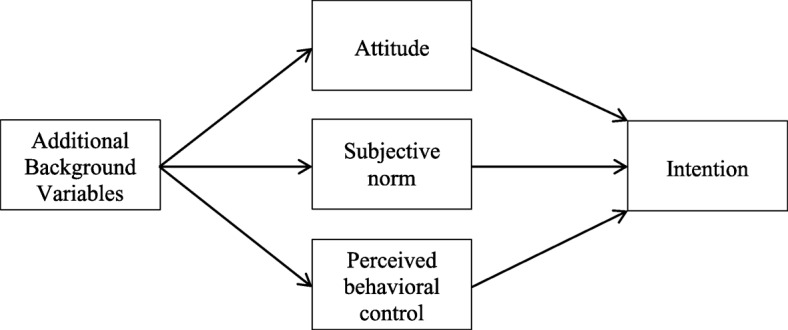
Theory of planned behavior with additional background variables as precursor variables Source: reproduced with permission from Springer, Hennessy et al., 2010

As Ajzen and Albarracin (2007) suggest that the TPB is open to the inclusion of further predictors, identifying a number of additional background variables within the context of the TPB may help improve the predictive power of the theory [16]. The theoretical framework for the TPB with additional background variables is presented in Fig. 1 [17]. There are a number of studies on intention to quit smoking among adults that incorporated extension predictors within the context of the TPB [8–12, 14]. Although these studies provided empirical support for the idea that inclusion of additional variables within the context of the TPB may help improve the predictive power of the theory, most of these studies have two major limitations. First, they used only individual level variables (e.g., past quit attempts and tobacco dependence) as additional predictors of the TPB. Macro or environmental level variables that may affect quitting intention should also be investigated and taken into account for the development of more comprehensive behavior interventions because such interventions hold promise for influencing large populations [18]. Second, using multiple regression models limited most of these studies to treat additional predictors of the TPB as control variables, rather than precursor variables. A recent study found out that there are substantial differences in the results between treating additional variables of the TPB as control variables and using additional variables of the TPB as precursor variables [17]. Hennessy et al. (2010) also suggest that the effects of all the additional predictors of the TPB on intention are assumed to be mediated by the TPB constructs (i.e., attitude, subjective norm, and perceived behavioral control) because additional predictors occur prior to the TPB constructs [17].

To our knowledge, there are only two studies on quitting intention among adults that treated additional predictors of the TPB as precursor variables [9, 10]. These two studies are quite different from other TPB studies listed above because they used structural equation modeling to examine the extended version of the TPB. The structural equation modeling made it possible to treat additional predictors of the TPB as precursor variables. However, Lee et al.’s (2006) study focused on testing the effects of individual level variables (i.e., independent self-construal and interdependent self-construal) on TPB constructs whereas Macy et al.’s (2011) study included two environmental factors (i.e., agreement with regulating smoking in public places and a comprehensive smoke-free air law) as extension predictors of the TPB [9, 10]. Since Macy et al.’s (2011) study is the only study that fulfills two limitations mentioned above, the present study attempted to reinforce their study by incorporating additional environmental variables (i.e., agreement with increasing taxes on cigarettes and the number of smokers among 5 closest friends) as precursor background variables of the TPB to predict quitting-related intentions and comparing the results between two population subgroups [10].

Using structural equation modeling, the present study attempted to incorporate various environmental variables as precursor background variables of the TPB. Over the past 25 years, smoke-free air laws have diffused quickly throughout the world [19]. The smoking restrictions in public places and worksites are originally intended to protect nonsmokers from secondhand smoke exposure. However, smoking restrictions have been shown to be highly effective in decreasing the number of cigarettes smoked and increasing intention to quit among smokers [20]. Further, smoke-free air law also has been shown to help smokers achieve successful smoking cessation [21]. Although there are empirical studies showing that smoke-free air laws are an effective strategy to reduce tobacco use and promote health of both smokers and nonsmokers, smoke-free air law can be less effective if it is not supported by target population. For example, the California Tobacco Control Program, which used social norm strategy for tobacco control activities, was evaluated by Zhang et al. (2010) and they found out that smokers who supported smoke-free air laws were more likely to report quit attempts and intention to quit under circumstance where smoke-free air law is enacted [22]. A number of recent studies also showed that smokers’ support for anti-smoking policies, such as cigarette tax increase and smoke-free air law, were cross-sectionally associated with intention to quit smoking under circumstance where tobacco control policy is implemented [23–26]. Therefore, the present study included comprehensive smoke-free air law and the extent of agreement with regulating smoking in public places and increasing taxes on cigarettes as precursor background variables of the TPB.

The observational learning plays an important part in the social cognitive theory. Performing new behaviors, such as smoking cessation, is most likely to be affected by family, friends, or media models [27, 28]. Since friends can serve as an important role model, friends’ smoking may also have tremendous effects on quitting intention among smokers. Friends’ smoking is also known to increase cigarette smoking by enhancing cigarette availability among smokers [29]. There have been many empirical studies showing that friends’ smoking is a primary determinant in predicting adolescent initiation and continuation of smoking [30]. However, it is less clear whether smoking status of friends influence continuation of smoking among adult smokers. To our knowledge, there were only three studies that investigated the relationship between friends’ smoking and continuation of smoking among young adult smokers and the results were inconsistent [31–33].

The aims of the present study are to incorporate environmental variables as precursor background variables of the TPB to predict quitting-related intentions (i.e., intention to take measures to quit and intention to quit) and to compare the results between two population subgroups (i.e., Texas residents aged 18 or older who were current cigarette smokers and Indiana University and Purdue University students aged 18 or older who were current cigarette smokers). The following research questions were addressed: (1) How do the TPB components (i.e., attitude, subjective norm, and perceived behavioral control) affect quitting-related intentions?; (2) How do the environmental variables indirectly affect quitting-related intentions through three components of the TPB?; and (3) If the results are different between two population subgroups (i.e., Texas adult smokers and university student smokers)?

## Methods

The present study consists of two sub-studies. Sub-study 1 examined intention to take measures to quit using extended version of the TPB among Texas residents who were current smokers and aged 18 or older. Sub-study 2 examined intention to quit using extended version of the TPB among Indiana University and Purdue University students who were current smokers and aged 18 or older. These two sub-studies analyzed different data sets and were conducted using the similar methodology for the comparison.

### Data

#### Sub-study 1

Sub-study 1 used the same dataset that was used in Macy et al.’s (2012) study [10]. In September and October of 2007, a quota sample of adults (aged 18 or more) was obtained from seven Texas cities representing a range of comprehensive smoke-free air laws using random digit dialing. Residents who reported living in one of the designated cities for at least 6 months were included in the sample because one of the primary interests was examining the effects of the city regulations. Data were collected by computer-assisted telephone interviews. The adult in the household whose birthday had most recently passed was selected for the interview. Up to three callbacks were conducted to reach that person. This procedure was continued until a target sample of 50 current or former smokers and 50 nonsmokers each from Austin, Dallas, El Paso, Fort Worth, Lubbock, and San Antonio and 100 current or former smokers and 100 nonsmokers from Houston was obtained. All participants reported their attitudes and perceptions of smoking, city they live in, and smoking ban in their home and car. Only those who reported current smoking (smoked at least 100 cigarettes in their lifetime and smoked every day or some days at the time of measurement) were included in the present study (*N* = 395).

#### Sub-study 2

During fall semester 2009, 87 instructors at Indiana University were asked for permission to execute a survey during their classes. Among 87 Indiana University instructors, 77 agreed to participate in the study. A total of 2215 Indiana University students were asked to complete a paper-and-pencil survey, and 2042 (92.2%) students decided to participate in the survey. The same procedure was replicated at Purdue University. Sixty five instructors at Purdue University were asked for permission to execute a survey during their classes. Among 65 Purdue University instructors, 54 agreed to participate in the study. A total of 1240 Purdue University students were asked to complete a paper-and-pencil survey, and 1165 (94.0%) students decided to participate in the survey. Among 3207 Indiana and Purdue University students (2042 in Indiana University and 1165 in Purdue University), only those who reported current smoking (smoked at least 100 cigarettes in their lifetime and smoked every day or some days at the time of measurement) were included in the present study (*N* = 379).

### Measures

#### TPB variables

Sub-study 1 and 2 used same questions regarding the TPB variables (i.e., intention, attitude, subjective norm, and perceived behavioral control). However, the target behaviors of interest were not identical between two studies (Sub-study 1 used “taking measures to quit smoking within the next 1 month” and Sub-study 2 used “quitting smoking within the next 6 months”). One thing noteworthy is that “taking measures to quit smoking” is similar to “quitting smoking” because the measures of taking measures to quit smoking include quitting smoking (e.g., go “cold turkey” or just quit suddenly without help, cut down gradually, stop with a friend, etc.) in addition to other methods of quitting smoking in Sub-study 1. In addition, since Sub-study 1 and 2 used different response scales for the TPB variables (Sub-study 1 used 5-point Likert scale and Sub-study 2 used 7-point Likert scale), the TPB variables were standardized to make them comparable between two studies [34, 35]. Intention to take measures to quit (or quit) was measured by asking participants how likely it is that they will try to take measures to quit (or quit) within the next 1 month (or 6 months). This item was rated on a 5-point (or 7-point) Likert scale ranging from very unlikely to very likely. Attitude towards taking measures to quit (or quitting) was measured by a question: “would your taking measures to not smoke cigarettes in the next month be good or bad?” This item was rated on a 5-point (or 7-point) Likert scale ranging from very bad to very good. Subjective norm was measured by asking participants how likely it is that most people who are important to them think they should try to take measures to quit (or quit) smoking within the next 1 month (or 6 months). This item was rated on a 5-point (or 7-point) scale ranging from very unlikely to very likely. To assess perceived behavioral control, participants answered one item asking how easy they think it would be for them to try to take measures to quit (or quit) smoking within the next 1 month (or 6 months). This item was rated on a 5-point (or 7-point) Likert scale ranging from very difficult to very easy.

#### Precursor background variables

All the precursor background variables used in Sub-study 1 and 2 were measured using exactly same questions except comprehensive smoke-free air law. To assess exposure to comprehensive smoke-free air law, Sub-study 1 divided participants into two groups on the basis of cities where they were living at the time of the interview (cities with a comprehensive smoke-free air law [Austin, El Paso, and Houston] and cities without a comprehensive smoke-free air law [Dallas, Fort Worth, Lubbock, and San Antonio]), whereas, in Sub-study 2, participants were divided into two groups on the basis of university they were attending (Indiana University or Purdue University). The new Tobacco Free Campus Policy (indoor and outdoor smoking ban) at Indiana University went into effect on January 1 in 2008, whereas Purdue University allowed students to smoke outdoors at a distance of minimum 30 ft from university facilities. The extent of agreement with regulating smoking in public places was measured by asking participants whether they agree with the following statement: “Regulation of smoking in public places is a good thing.” This item was rated on a 5-point Likert scale ranging from strongly disagree to strongly agree. The extent of agreement with increasing taxes on cigarettes was assessed by asking whether they agree with the following statement: “Taxes on cigarettes should be increased.” This item was also rated on a 5-point Likert scale ranging from strongly disagree to strongly agree. The number of smokers among 5 closest friends was measured by asking “How many of your 5 closest friends smoke cigarettes?” The response options ranged from 0 to 5. Demographic characteristics, such as age (continuous), sex (male or female), and race/ethnicity (non-Hispanic white or others) were also considered as covariates.

### Statistical analysis

The direct and indirect relationships of the variables were examined using the multiple group structural equation modeling. Mplus Version 5.21 was used to conduct the multiple group structural equation modeling analysis [36]. The parameters in the multiple group structural equation models were estimated using the maximum likelihood method. Indirect effects were tested using the Sobel method [37]. Because the Sobel test may incorrectly assume normality of the indirect effect especially when sample size is small, bootstrapped standard errors were used for the significance test of indirect effects [38]. The number of bootstrap draws was 1000. The order of variable entry in the model was based on the hypothesized theoretical framework. Using a multiple group procedure, the structural equation models for adult smokers and university student smokers were fitted simultaneously in order to examine possible differences between these two models. Three model fit indices were used to evaluate goodness of fit (i.e., chi-square, root mean square error of approximation [RMSEA], and comparative fit index [CFI]).

## Results

Table 1 presents descriptive statistics of participants. The mean age of Texas adult smokers was 46.08, whereas the mean age of university student smokers was 20.99. Nearly half of Texas adult smokers were non-Hispanic white (50.79%), whereas the majority of university student smokers were non-Hispanic white (83.51%). Although study participants were current smokers, the mean scores of attitude and subjective norm toward taking measures to quit smoking and quitting smoking were relatively high (positive). The extent of agreement with regulating smoking in public places was relatively higher than the extent of agreement with increasing taxes on cigarettes in both Sub-study 1 and 2.

**Table 1:** Descriptive statistics of participants in Sub-study 1 and Sub-study 2: Texas, United States, 2007 (sub-study 1) and Indiana, United States, 2009 (Sub-study 2)

Variable	Sub-study 1		Sub-study 2	
	Mean	(SD)	Mean	(SD)
Age	46.08	(15.64)	20.99	(4.51)
Sex (*n, %*)				
Male	184	(46.58)	195	(52.00)
Female	211	(53.42)	180	(48.00)
Race/ethnicity (*n, %*)				
Non-Hispanic white	193	(50.79)	314	(83.51)
Others	187	(49.21)	62	(16.49)
Intention^a^	2.99	(1.63)	3.19	(2.37)
Attitude^a^	4.22	(1.23)	5.24	(1.39)
Subjective norm^a^	3.92	(1.04)	4.58	(1.87)
Perceived behavioral control^a^	2.51	(1.56)	3.43	(2.11)
Number of smokers among 5 closest friends	2.94	(1.75)	3.08	(1.31)
Extent of agreement with regulating of smoking in public places^a^	3.29	(1.32)	3.45	(1.21)
Extent of agreement with increasing taxes on cigarettes^a^	1.94	(1.14)	2.01	(1.21)
Comprehensive smoke-free air law (*n, %*)				
Living in a city with comprehensive smoke-free air law	196	(49.62)	261	(68.87)
Living in a city without comprehensive smoke-free air law	199	(50.38)	118	(31.13)

The participants in Sub-study 1 were Texas residents aged 18 or older who were current cigarette smokers (*N* = 395) and the participants in Sub-study 2 were Indiana University and Purdue University students aged 18 or older who were current cigarette smokers (*N* = 379)

^a^Sub-study 1 used five-point Likert scale ranging from 1 to 5 and Sub-study 2 used seven-point Likert scale ranging from 0 to 6

**Fig. 2:**
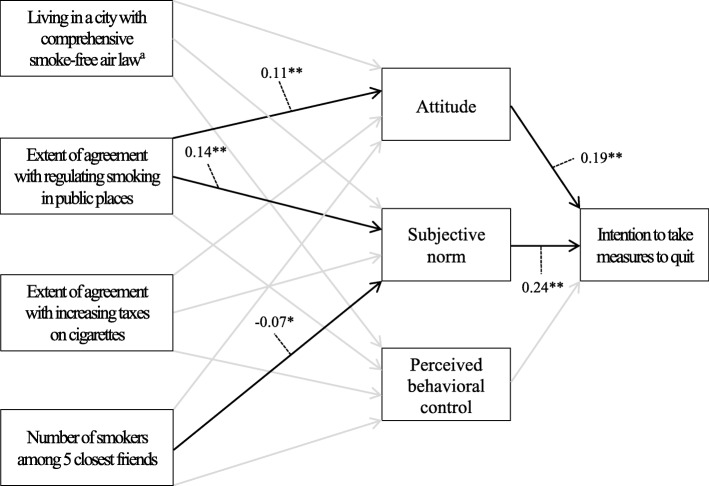
Path coefficients for a model predicting intention to take measures to quit among Texas adult smokers (*N* = 395): Texas, United States, 2007 Note: The effects of precursor background variables on three global constructs of the theory of planned behavior (i.e., attitude, subjective norm, and perceived behavioral control) were adjusted for age, sex, and race/ethnicity. a reference category is living in a city without comprehensive smoke-free air law. * *p* < .05, ** *p* < .01

**Fig. 3:**
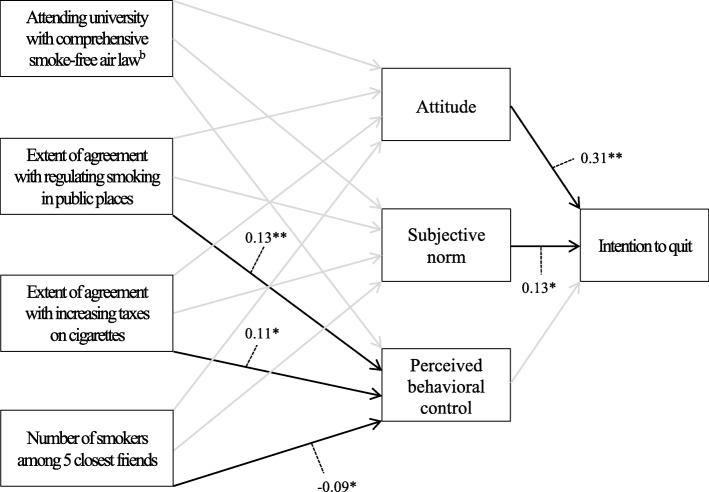
Path coefficients for a model predicting intention to quit among university student smokers (*N* = 379): Indiana, United States, 2009 Note: The effects of precursor background variables on three global constructs of the theory of planned behavior (i.e., attitude, subjective norm, and perceived behavioral control) were adjusted for age, sex, and race/ethnicity. b reference category is attending university without comprehensive smoke-free air law. * *p* < .05, ** *p* < .01

Figures 2 and 3 show path coefficients of the two structural equation models. A model shown in Fig. 2 (hereafter Model 1) predicts intention to take measures to quit among Texas residents aged 18 or older who were current cigarette smokers, and a model shown in Fig. 3 (hereafter Model 2) predicts intention to quit among Indiana University and Purdue University students aged 18 or older who were current cigarette smokers. The chi-square value was significant (χ^2^ = 26.575, df = 14, *p* < .05), RMSEA was 0.048, and CFI was 0.959, indicating that our two models are representing the data accurately [39, 40]. Attitude (Model 1: *β* = 0.19, standard error [SE] = 0.05, *p* < .01; Model 2: *β* = 0.31, SE = 0.05, *p* < .01) and subjective norm (Model 1: *β* = 0.24, SE = 0.05, *p* < .01; Model 2: *β* = 0.13, SE = 0.06, *p* < .05) were significantly associated with intention in both models. However, perceived behavioral control was not significantly associated with intention in both models. The effect of subjective norm on intention was not significantly different between two models, whereas the effect of attitude on intention was marginally significantly stronger in the case of Model 2 than Model 1 (*∆X*^2^ (1) = 2.792, *p* = .095).

Controlling for demographic characteristics (age, sex, and race/ethnicity), the comprehensive smoke-free air law did not have any significant effect on three global factors of the TPB (i.e., attitude, subjective norm, and perceived behavioral control) in either model. On the contrary, the extent of agreement with regulating smoking in public places was positively associated with attitude (*β* = 0.11, SE = 0.04, *p* < .01) and subjective norm (*β* = 0.14, SE = 0.04, *p* < .01) in Model 1 and perceived behavioral control (*β* = 0.13, SE = 0.04, *p* < .01) in Model 2. The extent of agreement with increasing taxes on cigarettes was also positively associated with perceived behavioral control (*β* = 0.11, SE = 0.05, *p* < .05) in Model 2. The number of smokers among 5 closest friends was negatively associated with subjective norm (*β* = − 0.07, SE = 0.03, *p* < .05) in Model 1 and perceived behavioral control (*β* = − 0.09, SE = 0.04, *p* < .05) in Model 2.

The indirect effects of precursor background variables on intention through three components of the TPB (attitude, subjective norm, and perceived behavioral control) were also examined (Table 2). The extent of agreement with regulating smoking in public places had positive indirect effects on intention to take measures to quit through attitude (*β* = 0.02, *p* < .01) and subjective norm (*β* = 0.03, *p* < .01) in Model 1. Although there was no significant association between the extent of agreement with regulating smoking in public places and subjective norm in Model 2, the extent of agreement with regulating smoking in public places had significant positive indirect effect on intention to quit through subjective norm (*β* = 0.01, *p* < .05). The number of smokers among 5 closest friends had negative indirect effect on intention to take measures to quit through subjective norm (*β* = − 0.02, *p* < .05) in Model 1. The comprehensive smoke-free air law and the extent of agreement with increasing taxes on cigarettes did not have any significant indirect effect on intention through three global factors of the TPB in either model.

**Table 2:** Significant indirect effects of precursor background variables on quitting-related intentions: Texas, United States, 2007 (Model 1) and Indiana, United States, 2009 (Model 2)

Significant indirect effect			Beta coefficient
Model 1			
Extent of agreement with regulating smoking in public places	➔ Attitude	➔ Intention	−0.02**
Extent of agreement with regulating smoking in public places	➔ Subjective norm	➔ Intention	−0.03**
Number of smokers among 5 closest friends	➔ Subjective norm	➔ Intention	−0.02*
Model 2			
Extent of agreement with regulating smoking in public places	➔ Subjective norm	➔ Intention	−0.01*

* *p* < .05, ** *p* < .01

## Discussion

The present study incorporated environmental variables as precursor background variables of the TPB to predict quitting-related intentions and compared the results between Texas adult smokers and university student smokers. This study attempted to reinforce Macy et al.’s (2012) study by: (1) incorporating additional environmental variables as precursor background variables of the TPB to predict quitting-related intentions; (2) comparing the results between two population subgroups (i.e., Texas adult smokers and university student smokers); and (3) using a formal significance test of the indirect effects to examine whether precursor background variables indirectly affect quitting-related intentions through three global constructs of the TPB.

The results of both Sub-study 1 and 2 showed that attitude and subjective norm were significantly associated with quitting-related intentions, whereas perceived behavioral control was not associated with quitting-related intentions. This finding is inconsistent with a recent review of the application of the TPB to health-related behaviors showing that attitude was the strongest predictor of intention and the magnitude of the effect of perceived behavioral control on intention was almost similar to that of attitude [41]. The non-significant effect of perceived behavioral control on quitting-related intentions may be due to difficulty of measuring perceived behavioral control over addictive behaviors. Since smoking cigarettes is an addictive behavior, a questionnaire for perceived behavioral control could have measured perceived lack of control (perceived difficulty) over quitting smoking and it is suggested that perceived behavioral control and perceived difficulty are unidimensional or distinct [14, 42, 43]. In addition, Ajzen (2002) emphasizes that perceived behavioral control may reflect self-efficacy as well as controllability and whether or not perceived behavioral control should be assessed by one or two components is an empirical question [44]. On the contrary, the measure of perceived behavioral control was assessed by a single question asking participants how easy they think it would be for them to try to take measures to quit (or quit) smoking in Sub-study 1 and 2.

The results of the present study also showed that the effects of subjective norm on quitting-related intentions were not significantly different between Sub-study 1 and 2, whereas the effects of attitude on quitting-related intentions were marginally significantly stronger in the case of university student smokers than Texas adult smokers. One possible interpretation of this results is that college students’ smoking behavior is shown to be strongly related to the images of being confident, mature, fashionable, sophisticated, or cool [45, 46], which may be closely related to their attitude towards quitting-related behaviors. As the effects of attitude on quitting-related intentions were stronger in university student smokers than Texas adult smokers, public health practitioners and researchers may need to focus more on attitudes in developing smoking cessation intervention programs in college or university settings.

The results of this study showed that the extent of agreement with regulating smoking in public places had positive indirect effects on quitting-related intentions through subjective norm in both Sub-study 1 and 2. Our results also showed that the extent of agreement with regulating smoking in public places had positive indirect effects on intention to take measures to quit through attitude among Texas adult smokers only. These results are in line with previous studies showing that smokers’ support for smoke-free air law was positively related to intention to quit smoking [22–24, 26].

On the contrary, the results of the present study showed that the extent of agreement with increasing taxes on cigarettes did not have any significant indirect effect on intention in either model. It has been shown that the support for increasing tax on cigarettes is far higher if the extra revenue is used to support quitting smoking and promote healthy lifestyle [47, 48]. Moreover, Wilson et al. (2010) found out that although most smokers reported that the current tobacco tax is “too high”, the majority of smokers supported increase in tax on tobacco if the tax revenue is used to promote healthy lifestyle [25]. They also found out that the support for increasing a dedicated tobacco tax was significantly positively associated with intention to quit. The non-significant indirect effect of the extent of agreement with increasing taxes on cigarettes on quitting-related intentions may be due to the questionnaire used in the present study. In Sub-study 1 and 2, the questionnaire asked participants about their agreement with increasing taxes on cigarettes but did not mention how the tax revenue would be used.

The number of smokers among 5 closest friends had negative indirect effect on intention to take measures to quit through subjective norm among Texas adult smokers. This result is in line with social cognitive theory suggesting that performing new behaviors, such as smoking cessation, is most likely to be affected by family, friends, or media models [27, 28]. This result is also consistent with Etcheverry and Agnew’s (2008) study showing that both friends’ smoking and subjective norm towards smoking were significantly associated with smoking behavior among young adult smokers [31]. However, in our study, the number of smokers among 5 closest friends did not have any significant indirect effect on intention to quit through three components of the TPB among university student smokers. The mean age of university student smokers in our study was 20.99, which means they may have experienced the significant changes in social relationships that often occur right after graduation from high school. Therefore, it is possible that university students in our study recalled their high school friends who could not spend much time together when trying to answer a questionnaire regarding friends’ smoking. There were three studies that examined the relationship between friends’ smoking and smoking behavior among young adult smokers [31–33]. Among these studies, Etcheverry and Agnew’s (2008) study was the only study that asked participants to list friends with whom they spent the most time when assessing friends’ smoking and their study was the only study that showed significant association between friends’ smoking and smoking behavior among young adult smokers [31]. Future studies on the relationship between friends’ smoking and smoking behavior among young adult smokers should also ask participants about friends who spent most time together when measuring friends’ smoking.

The findings of this study are subject to several limitations. First, the fact that participants were exposed to the smoke-free air law for a different amount of time may have influenced our findings. However, it was difficult to resolve this methodological problem because of data constraints and unpredictable process of policymaking. Second, most of the variables used in this study were self-reported, which may cause respondent bias, interview bias, or recall bias. Third, due to the cross-sectional design, causal relationships among study variables cannot be determined. Some caution is required in interpreting the results of this study. Fourth, the different results between Sub-study 1 and 2 may also be caused by different intentions analyzed in each study (Sub-study 1 analyzed intention to take measures to quit within the next 1 month and Sub-study 2 analyzed intention to quit within the next 6 months). A comparison of the TPB models that use exactly same type of intention would be more desirable.

## Conclusions

The results of this study may contribute to the literature by providing valuable information suggesting that environmental variables need to be considered as precursor background variables of the TPB to predict quitting-related intentions. This study underlines the importance of addressing smokers’ support for smoke-free air law when designing future smoking interventions for both Texas adults and University students. For Texas adults, it is also suggested that promoting smoking cessation among close friends may be one of effective ways to increase their intention to quit smoking. The finding of this study also confirms the importance of taking policy and environmental approaches to promoting public health in a community.

## Abbreviation

TPB: Theory of Planned Behavior
